# Castleman’s Disease, a Case report

**Published:** 2011

**Authors:** Ranga Reddy, Ankit Singhania, Ajay George, Ranjan Kumar, Sanjay Kumar

**Affiliations:** 1 *Department of otorhinolaryngology, Kamineni Institute of Medical Sciences, India*; 2 *Department of otorhinolaryngology, SBKS Medical Institute and Research Centre, India*; 3 *Department of otorhinolaryngology, SBKS Medical Institute and Research Centre, India*; 4 *Department of otorhinolaryngology, Kamineni Institute of Medical Sciences, India*; 5 *Department of otorhinolaryngology, Kamineni Institute of Medical Sciences, India*

**Keywords:** Castleman’s disease, Cervical, Lymphadenopathy

## Abstract

**Introduction:**

Castleman's disease is a rare lymphoproliferative disorder which may be confused with other causes of lymphadenopathy.

**Case Report:**

Here we report a case of unicentric Castleman's disease presenting with cervical lymphadenopathy. The patient was treated with complete surgical excision of lesion and was disease free at the time of reporting this article. This case has been reported for its rarity.

**Conclusion:**

Though castleman’s disease is a relatively rare entity it should be strongly considered in the differential diagnosis of cervical lymphadenopathy.

## Introduction

Castleman’s disease is a rare, poorly understood, lymphoproliferative disorder. It is also known as angiofollicular lymph node hyperplasia or giant lymph node hyperplasia (1). There are only about 400 cases reported up to now (2). This case has been reported for its rarity. 

## Case report

A 46 year old female patient presented with history of swelling on left side of the neck for two years. The painless swelling was gradually increasing in size. There was no history of constitutional symptoms like fever, weight loss and night sweats. There was no history of pain in the throat, dysphagia, dyspnea change of voice and exposure to tuberculosis either.

General examination of the patient including ear, nose and throat was normal. Neck examination showed a firm, non-warm, non-tender, non-reducible swelling, in the posterior triangle of neck on the left side measuring 7cms in its greatest dimension which was freely mobile in all directions. 

There was no movement on deglutition or protrusion of tongue. There were no generalized lymphadenopathy, hepatomegaly or splenomegaly.

All routine lab tests like CBP, CUE, RBS, and blood urea and serum creatinine were within normal limits. ESR was elevated at 50 mm in the first hour. Fine needle aspiration cytology showed inconclusive findings. Surgical excision biopsy was done under general anesthesia. The mass was removed and sent for histopathological examination. Intra-operative and post-operative period were uneventful. Histopathology showed typical picture of hyalinized follicle surrounded by lymphocytes arranged in “onion skin” layer like pattern in the lymph node, leading to a conclusion of Castleman’s disease, hyaline-vascular type. The patient was followed up and at the time of reporting this article has been symptom free for 8 months. 

**Fig 1:**
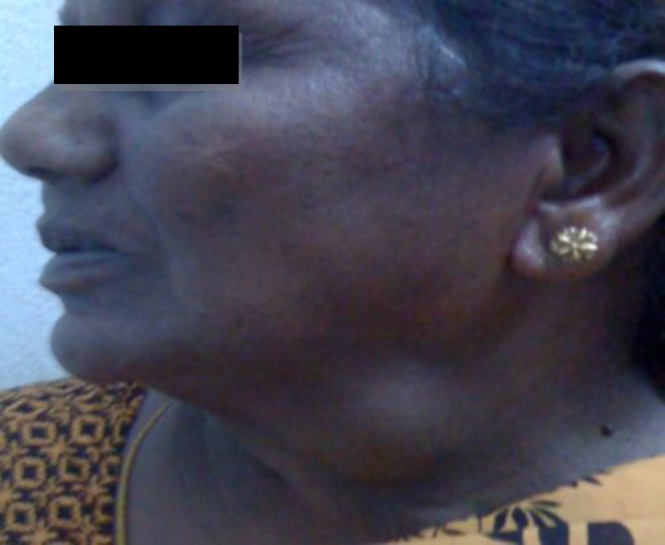
Clinical view of the patient

## Discussion

Castleman’s Disease was originally described by Dr. Benjamin Castleman’s in 1954. Three types of Castleman’s disease have been identified. These are hyaline-vascular (HV) type (about 90% of individuals), plasma cell (PC) type, and mixed variant (MV) type. There are 2 different clinical entities: the unicentric type which only one anatomic lymh node affected and the multicentric type characterized by generalized lymphadenopathy, constitutional symptoms and more aggressive clinical course (1). Unicentric Castleman’s disease occurs at a relatively younger average age of 37 years. Though mediastinal nodes are the commonest presenting feature, cervical lymphadenopathy, as in our case occupies the second place. 

It is a well documented fact that the HV type is the commonest type (1). Patients of unicentric disease usually do not have constitutional symptoms, as in our case (3).

Surgical removal of the affected lymph node is the treatment of choice. Good results have been reported in literature with surgical excision (4). If surgical removal isn't possible, in cases that the lymph node is difficult to get to, radiation therapy (4) may be an effective way to destroy the affected tissue. In the plasma cell and multicentric types, corticosteroids (5), chemotherapeutic drugs, monoclonal antibodies and immunomodulators may be used (6).

This approach is usually reserved for patients refusing surgery or having multicentric disease. Long term follow up of patients of Castleman’s disease shows very low or nil recurrence in cases which have been successfully treated. However there should be a constant look out for malignant sequel (4). 

## Conclusion

This case report is presented for its rarity. Cervical lymph nodes are involved by Castleman’s disease and may be confused with other common causes of cervical lymphadenopathy like tuberculosis and nodal secondaries. Surgical removal of the tumors in the unicentric hyaline-vascular type of Castleman’s disease is the treatment of choice. 
